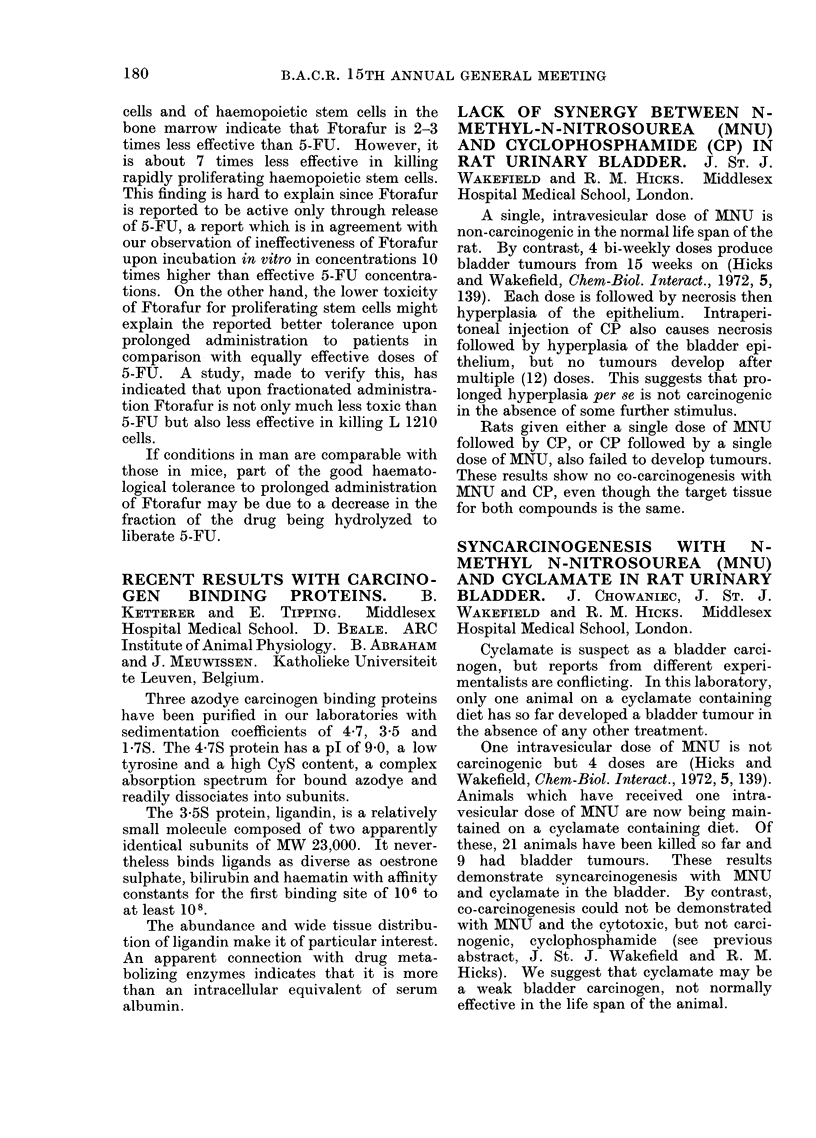# Proceedings: Recent results with carcinogen binding proteins.

**DOI:** 10.1038/bjc.1974.155

**Published:** 1974-08

**Authors:** B. Ketterer, E. Tipping, D. Beale, B. Abraham, J. Meuwissen


**RECENT RESULTS WITH CARCINOGEN BINDING PROTEINS.** B. Ketterer and E. Tipping. Middlesex Hospital Medical School. D. Beale. ARC Institute of Animal Physiology. B. Abraham and J. Meuwissen. Katholieke Universiteit te Leuven, Belgium.

Three azodye carcinogen binding proteins have been purified in our laboratories with sedimentation coefficients of 4·7, 3·5 and 1·7S. The 4·7S protein has a pI of 9·0, a low tyrosine and a high CyS content, a complex absorption spectrum for bound azodye and readily dissociates into subunits.

The 3·5S protein, ligandin, is a relatively small molecule composed of two apparently identical subunits of MW 23,000. It nevertheless binds ligands as diverse as oestrone sulphate, bilirubin and haematin with affinity constants for the first binding site of $10^6$ to at least $10^8$.

The abundance and wide tissue distribution of ligandin make it of particular interest. An apparent connection with drug metabolizing enzymes indicates that it is more than an intracellular equivalent of serum albumin.